# Effect of melatonin on developmental competence, mitochondrial distribution, and intensity of fresh and vitrified/thawed in vitro matured buffalo oocytes

**DOI:** 10.1186/s12958-024-01209-7

**Published:** 2024-04-05

**Authors:** Omaima Mohamed Kandil, Samar Mahfouz Abd El Rahman, Rania S. Ali, Esraa Aly Ismail, Nehad M. Ibrahim

**Affiliations:** 1 Department of Animal Reproduction & Artificial Insemination, Veterinary Research Institute, National Research Centre, Cairo, Egypt; 2 Accredited (ISO 17025) Embryo and Genetic Resources Conservation Bank in National Research Centre (NRC), Cairo, Egypt; 3 Zoology and Entomology Department, Faculty of Science, Helwan University, Cairo, Egypt

**Keywords:** Buffalo, In-vitro embryo development, Melatonin, Mitochondria, Vitrification

## Abstract

Background: In livestock breeding, oocyte cryopreservation is crucial for preserving and transferring superior genetic traits. This study was conducted to examine the additional effect of melatonin to maturation and vitrification media on the in vitro developmental capacity, mitochondrial distribution, and intensity of buffalo oocytes. The study involved obtaining ovaries from a slaughterhouse and conducting two phases. In the first phase, high-quality oocytes were incubated in a maturation medium with or without 10^−9^M melatonin for 22 h (at 38.5°C in 5% CO_2_). Matured oocytes were fertilized in vitro and cultured in SOF media for seven days. In the second phase, vitrified in vitro matured oocytes were stored in vitrified media (basic media (BM) containing a combination of cryoprotectants (20% Ethyl Glycol and 20% Dimethyl sulfoxide), with or without melatonin, and then stored in liquid nitrogen. Normal vitrified/thawed oocytes were fertilized in vitro and cultured as described. Finally, the matured oocytes from the fresh and vitrified/thawed groups, both with and without melatonin, were stained using DAPI and Mitotracker red to detect their viability (nuclear maturation), mitochondrial intensity, and distribution using a confocal microscope. The study found that adding 10^−9^M melatonin to the maturation media significantly increased maturation (85.47%), fertilization rate (84.21%)cleavage (89.58%), and transferable embryo (48.83%) rates compared to the group without melatonin (69.85%,79.88%, 75.55%, and 37.25% respectively). Besides that, the addition of melatonin to the vitrification media improved the recovery rate of normal oocytes (83.75%), as well as the cleavage (61.80%) and transferable embryo (27.00%) rates when compared to the vitrified TCM group (67.46%, 51.40%, and 17.00%, respectively). The diffuse mitochondrial distribution was higher in fresh with melatonin (TCM + Mel) (80%) and vitrified with melatonin (VS2 + Mel groups) (76.70%), Furthermore, within the same group, while the mitochondrial intensity was higher in the TCM + Mel group (1698.60) than other group. In conclusion, Melatonin supplementation improves the developmental competence and mitochondrial distribution in buffalo oocytes in both cases(in vitro maturation and vitrification).

**Supplementary Information:**

The online version contains supplementary material available at 10.1186/s12958-024-01209-7.

## Introduction

Over the past few years, ART (Assisted Reproductive Techniques) such as embryo transfer, artificial insemination, IVEP (in vitro embryo production), and cryopreservation have gained popularity. These techniques potentially improve reproductive efficiency, increase the production of superior animals, increasing the offspring from selected females, and also help in reduction of generation intervals in buffaloes [1]. Producing high-quality embryos from cows through in vitro production is a widely used and effective technique [2]. Maturation of the oocyte is still one of the most critical parts of the in vitro embryo production process. However, buffalo oocytes matured in vivo have higher quality and development than those matured in vitro [3]. Excessive reactive oxygen species (ROS) lead to harmful effects on cell components like fragmentation in DNA, oxidation in protein, peroxidation in lipid, and mitochondrial damage [4]. Antioxidants are included in in-vitro maturation (IVM) media to enhance oocyte maturation and embryo development [5]. Melatonin, also known as Mel, is recognized for its powerful antioxidant and anti-apoptosis properties. It can protect DNA and other components from damage and reduce oxidative stress caused by ROS in all cells [6]. Melatonin can improve the development of in vitro embryos in bovine [7], mice [8], pigs [9], dromedary camel [10], and humans [11]. Also, Mel can protect cumulus cells from nuclear fragmentation while lowering ROSlevels in bovine oocytes [12]. Melatonin can also improve the expansion of cumulus cell, the maturation rate in nucleus, cleavage and blastocyst rates in bufflo oocytes [13].

Oocyte cryopreservation can be achieved through slow freezing or vitrification, but the conventional slow-freezing method for bovine oocytes and embryos frequently causes cellular injury due to osmotic stress and ice crystallization [14]. Cryopreservation through vitrification is a highly efficient method that can minimize damage caused by low temperatures and lessen the impact of osmotic shock on embryos. The success of the cryopreservation process depends on a fine balance between the freezing rate and the use of cryoprotective agents (CPAs) to help in prevent the dehydration of cells and the formation of ice within them [15]. In the vitrification of buffalo oocytes, the most common cryoprotectants used are ethylene glycol and Dimethyl sulfoxide [16]. Addition of melatonin to the vitrification media of mice oocytes improves the oocyte quality after warmed by reducing oxidative stress which maintains oocyte mitochondrial function [17]. The addition of 10^-9^M Mel improved the developmental competence of parthenogenetic vitrified/ warmed in oocyte mice [18].

Mitochondria have critical roles in cellular energy metabolism for the majority of oocyte processes [19]. Oocyte quality is influenced by mitochondria; the location of mitochondria and the amount of mitochondrial transcripts are two key indications of oocyte cytoplasmic development [20]. The mitochondrial system is highly sensitive to temperature changes [21].Vitrification can affect mitochondrial function and increase ROS levels, leading to a loss of subsequent embryo development ability [22], as well as in people [23], mice [24], porcine [25], and bovine [26]. Melatonin can recover the balance of mitochondrial function and improve the meiotic maturation in vitrified mice oocytes [27]. Also could improve mitochondrial function and quality of in vitro matured aged mice oocytes [28].

Our research has shown that adding melatonin to the vitrification media can enhance the developmental competence and mitochondrial intensity of camel oocytes after being thawed [10]. This study is the first one to investigate the effects of melatonin on both maturation and vitrification media. Our goal was to examine how melatonin affects the morphology, developmental competence, mitochondrial intensity, and distribution of fresh in-vitro matured and/or vitrified/thawed in-vitro matured buffalo oocytes.

## Materials and methods

Chemicals and media used in this study were purchased from Sigma-Aldrich (Sant. Louis, MO, USA) unless otherwise mentioned.

### Collection of oocytes and IVM

The laboratory received buffalo ovaries from the slaughterhouse in a thermos filled with warm normal saline solution at 37 °C (0.9% NaCl + 100 IU penicillin and 100μg/ml streptomycin). The ovaries were cleaned several times in warmed saline solution, then placed in a water bath at 37 °C till the oocytes were ready to be aspirated. An 18-gauge needle connected to a sterile syringe containing aspiration and washing medium consists of PBS, 4 mg/ml BSA and 50 μg/ml gentamicin to aspirate COCs (cumulus oocyte complexes) from follicles 2–8 mm in diameter. Thereafter, follicular content was transferred to Falcon tube and left for 10–15 min to settle at 37 °C. The COCs were examined under a stereo microscope and rinsed for 3-times in oocyte maturation medium. According to Kandil et al*.* [29], buffalo oocyte quality was assessed. Depending on the cumulus investment and equally granulated ooplasm, there were four groups of COCs under a stereomicroscope (90 x) as follows:

- a-Excellent: Oocytes with at least five layers of fully developed cumulus cells (CC) and evenly granulated dark ooplasm.
- b- Good: One to four layers of cumulus cells and evenly granulated dark cytoplasm.
- c- Fair: Oocytes are incompletely encircled by cumulus cells, and the ooplasm has little granulation.
- d-Denuded: Oocytes were covered by zona pellucida and had no cumulus cells.

Excellent and good oocytes transferred to in vitro maturation medium (TCM-199 + 10% FCS + 10 μg/ml FSH + 50 μg/ml gentamicin) with or without 10 ^−9^ M Mel. (M5250, Sigma). The COCs were in vitro matured in an incubator at 38.5°C in a humidified atmosphere with 5% CO2 for 22 h. After 22 h from incubation, the cytoplasmic maturation of oocytes assessed according to the degree of cumulus expansion, according to the extent of cumulus-cell development, the cytoplasmic maturation of buffalo oocytes was evaluated and divided into 4-grades according to Kandil et al*.* [29]:

- G0: Without expansion.
- GI: With slight expansion.
- GII: Moderate expansions.
- GIII: Full expansionII.

Maturation of oocytes’ nuclear determined by the presence of the 1^st^ polar body (Pb) in the perivitelline space. The 1^st^ Pb was detected using an inverted microscope at 20X magnification. The expansion rate was obtained by dividing the number of oocytes per grade by the total number of oocytes and then the results were multiplied by 100. The nuclear maturation rate (M II) was calculated by dividing the number of mature oocytes with 1^st^ Pb on the total number of oocytes, and the results were multiplied by 100.

### In vitro embryo production

To prepare for fertilization, matured oocytes with full cumulus expansion was washed in a fertilization medium, specifically Fert-TALP [30] supplemented with 6 mg/ml BSA. Meanwhile, the frozen semen straw was thawed in a 37°C water bath for 30 s. The spermatozoa were then suspended in 5 ml of sperm-TALP medium, containing 10μg/ml heparin and 4 mg/ml BSA, before undergoing centrifugation for 10 min at 1800 rpm (344 g) in 15 ml conical tubes. After removing the supernatant, the sperm pellet was resuspended in Fert-TALP media for 3 ml and centrifuged for 5 min at 1800 rpm. Once centrifugation was complete, the supernatant was removed, and the sperm pellet was suspended in Fert-TALP supplemented with 20 μM penicillamine, 10μM/ml hypotaurine, and 1µM/ml epinephrine (PHE) + 6mg/ml BSA.. Next, the sperm suspension was placed into a 4-well culture plate with the oocytes and covered with warm 200 ul mineral oil. The concentration of sperm was adjusted to 1 × 10 ^6^ sperm/ml. The sperm and oocytes were co-incubated for 18 h at 38.5°C and 5% CO_2_ in humid air.

$$\mathrm{Rate}\;\mathrm{of}\;\mathrm{fertilization}\hspace{0.17em}=\hspace{0.17em}\text{No}.\;\mathrm{of}\;\mathrm{oocytes}\;\mathrm{with}\;2^{\mathrm{nd}}\;\mathrm{polar}\;\mathrm{body}\hspace{0.17em}\times\hspace{0.17em}100\hspace{0.17em}\div\hspace{0.17em}\mathrm{Total}\;\mathrm{no}.\;\mathrm{of}\;\mathrm{matured}\;\mathrm{oocytes}$$

The zygotes were decomulated using multiple pipetting and then washed three times and placed in a (modified synthetic oviduct fluid, mSOF) solution with 5 mg/ml of BSA and 50 μg/ml of gentamycin. Then, they incubated at 38.5°C with 5% CO2 in a humidified environment. The embryos were observed using an inverted microscope to determine their cleavage rate and development to the morula and blastocyst stages on Days 5 and 7. We change the mSOF medium every 2 days. Also, we checked the rate of cleavage and embryo development on days 2, 5, and 7. Then the detection of the cleavage rate and transferable embryos (morula and blastocyst) is calculated as follows:

- a-Cleavage rate was obtained by dividing the number of cleaved zygotes by the total number of fertilized oocytes with the second polar body multiplied by 100.
- b-The transferable embryo (morula and blastocyst) was obtained by dividing the number of morula and blastocyst by the total number of cleaved zygotes multiplied by 100 [16].

#### Oocytes vitrification

Matured oocytes without cumulus cells with 1^st^ polar body were placed in basic media (BM). This medium consists of 9.5 ml TCM-199, 0.5 ml FCS, and 50 μg/ml gentamicin. Afterward, oocytes were placed in vitrification solution one (VS,1 consists of BM, 10% Ethyl Glycol (EG), and 10% Dimethyl sulfoxide (DMSO)) for 1 min. Then oocytes are transferred to vitrification solution two (VS2, contains BM, 20% EG, and 20% DMSO) for 30 s. Finally, it loaded in a VS2 medium [16].

#### Oocytes loading

Oocytes were loaded into 0.25ml of French straw using a micro-classic pipette (Karl Hecht No. 558). First loading, about 20 ul hold medium consisting of BM + 0.5 M sucrose was drawn and separated from the oocytes in the vitrification solution (25 ul) using an air bubble chamber from two sides then drawn hold medium to the end of the straw. About 25–30 oocytes and polyvinyl powder were loaded into the straw to seal the loaded straw. The straw was exposed to liquid nitrogen (LN2) vapor for 10 s. then plunged into LN2 and stored for 7 days [16].

#### Oocytes warming

After 7 days of being vitrified, straws containing vitrified oocytes were warmed in a 37°C water bath for 10–15 s. The straws were shaken to mix the vitrification solution and holding medium with oocytes, then the warmed oocytes were transferred to a new thawing medium that contained sucrose. The sucrose was gradually diluted in a three-step process, starting with a concentration of 0.5 M, followed by 0.3 M, and finally 0.17 M, with 1 min of equilibration time in each solution. The oocytes were washed thrice with fresh BM [16].

#### Evaluation of morphological changes

Recovered vitrified oocytes were evaluated under an inverted microscope and the recovered vitrified oocyte rate equaled the number of recovered oocytes divided by the number of vitrified oocytes multiplied by 100.

Normal oocytes were spherical and symmetrical, showing no evidence of damage to the membrane, swelling, degeneration, or fragmented or shrinking cytoplasm. Conversely, abnormal oocytes exhibited a ruptured or cracked zona pellucida (ZP), fragmented or shrunk cytoplasm, and signs of degeneration or leakage of cellular contents. $\mathrm{Morphologically}\;\mathrm{normal}\;\mathrm{oocyte}\;\mathrm{ rate} =\frac{\text{No}.\;\mathrm{of}\;\mathrm{normal}\;\mathrm{vitrified}\;-\;\mathrm{thawed}\;\mathrm{oocytes}\;\mathrm{with}\;1\mathrm{st}\;\mathrm{polar}\;\mathrm {body}\;x\;100}{\mathrm{Total}\;\mathrm{no}.\;\mathrm{of}\;\mathrm{recovered}\;\mathrm{invitro}\;\mathrm{matured}\;\mathrm{vitrified}\;/\;\mathrm{thawed}\;\mathrm{oocytes}}$ [16].

#### Detection of mitochondrial distribution and intensity:

In-vitro matured buffalo oocytes either fresh or vitrified/warmed were stained using Mitochondrion specific fluorescent probe, Mito Tracker Red FM (thermo fisher). Confocal microscopy (Zeiss LSM 710) was used to determine mitochondrial distribution in the oocytes according to manufacturer instructions. In-vitro matured buffalo oocytes fresh or vitrified/ warmed were incubated with a final concentration of 500 nM Mito Tracker Red FM in PBS for 30 min at 37 ºC in the incubator. Oocytes were washed twice in PBS and co-incubated with PBS containing five µg/mL DAPI to counterstain the nucleus and detect the 1^st^ polar body in matured oocytes (MII). DAPI detection in excitation 358 mm and emission in 4601mm wavelength using a confocal microscope in 20X magnification. Oocytes were then washed and mounted in PBS and were visualized in a glass bottom culture plate 12 mm diameter (thermo fisher) using a confocal microscope (Zeiss 710). The Mito Tracker red fluorescence was observed using an argon laser in a 581nm excitation line and 644 nm emission filters. One optical section was examined for each oocyte, in the plane where the nucleus was visible. The determination of the mitochondrial distribution was as follows: peripheral mitochondrial distributions were in the oocytes in which no mitochondria were found at the center of the oocyte; semi-peripheral distributions were in those with an inhomogeneous distribution of mitochondria in the inner region of the oocyte; and diffuse distributions were those with a uniform distribution of mitochondria across the entire inner region of the oocyte cytoplasm. The mitochondrial intensity is automatically detected through the software of a confocal microscope [16]. The experiment was replicated three times, with a group of 10 oocytes in each replicate.

#### Experimental design

### Experiment 1: effect of melatonin addition to maturation media on maturation rate and developmental competence of buffalo oocytes

The total number of buffalo ovaries used in this experiment was 290 and excellent (*n* = 283) and good (*n* = 240) oocytes were used for IVM.

Two groups of in vitro matured oocytes The control group (TCM *n* = 289) underwent maturation without any melatonin treatment, while the treated group (TCM + Mel *n* = 234) was exposed to 10 ^−9^ M melatonin. After maturation, the oocytes were fertilized in-vitro and cultured using the abovementioned method. This was done to determine the rate of maturation, cleavage, and transferable embryo.

### Experiment 2: effect of melatonin on morphological changes of vitrified/warmed in-vitro matured buffalo oocytes

The total number of buffalo ovaries used in this experiment was 322 and excellent (*n* = 345) and good (*n* = 360) oocytes were used for IVM.

Excellent and good oocytes were in-vitro matured as experiment 1. In-vitro matured oocytes with 1^st^ PB (526) were used for vitrification in three groups: vitrified matured oocytes without Mel as control (VTCM *n* = 176), vitrified matured oocytes with Mel in maturation medium (VTCM + Mel *n* = 182) and vitrified matured oocytes with Mel in vitrification medium (VS2 + Mel *n* = 168) all groups were vitrified using 20%Ethelyn Glycol + 20% Dimethyl sulfoxide as described previously The vitrified in vitro matured buffalo oocytes were thawed after one week of vitrification for morphological evaluation.

### Experiment 3: effect of melatonin on developmental competence of vitrified/thawed in vitro matured buffalo oocytes

Normal oocytes used for this experiment were 374. VTCM (*n* = 112), VTCM + Mel (*n* = 128) and VS2 + Mel (*n* = 134).

Morphologically normal vitrified/thawed oocytes were subjected to in vitro fertilization as described above. At the end of the fertilization period, the presumptive zygotes were cultured in mSOF medium for 7 days. The cleavage and transferable embryo rate were evaluated

### Experiment 4: effect of melatonin on mitochondrial distribution and intensity of fresh and vitrified/warmed in-vitro matured buffalo oocytes

A total number of 150 fresh in-vitro matured and in-vitro matured vitrified / thawed warmed buffalo oocytes were stained using a Mitochondrion-specific fluorescent probe, Mito Tracker Red FM (Thermo Fisher). Confocal microscopy (Zeiss LSM 710) was used to determine mitochondrial distribution and intensity in the oocytes as described previously. DAPI stain was used to counterstain the nucleus and enable the determination of nuclear maturation.

### Statistical analysis

The IBM SPSS software program version 25.0 was used to analyze the data. The data was described using numbers and percentages. The significance of differences was determined using an independent t-test, analysis of variance (ANOVA), followed by a post hoc test (*P* ≤ 0.01). The chi-square test was used for categorical variables.

## Results:

### Experiment 1: effect of melatonin addition in in-vitro maturation media on maturation rate and developmental competence of buffalo oocytes

#### Effect of melatonin addition in in-vitro maturation media on cytoplasmic maturation (cumulus expansion rate) of matured buffalo oocytes

Results in Table 1 demonstrate that the addition of 10^-9^M melatonin to maturation media resulted in a significant (*P*<0.05) increase in the percentage of oocytes with GIII cumulus expansion by (61.11%) when compared with oocytes cultured without melatonin media (39.44%). The TCM group showed a significant increase in cumulus expansion rate GII, GI, and G0 (23.18%, 20.76%, and 16.60 %, respectively) when compared with TCM +Mel (18.80%, 12.82 %, and 7.26%, respectively).

**Table 1:** Effect of melatonin addition to maturation media on cytoplasmic maturation of buffalo oocytes

**Media**	**No. of oocytes**	**Cumulus expansion grade**											
		**GӀӀӀ**			**GӀӀ**			**GӀ**			**G0**		
		**No.**	**Mean ± SE**	**%**	**No.**	**Mean** ** ± SE**	**%**	**No.**	**Mean ± ** **SE**	**%**	**No.**	**Mean** ** ± SE**	**%**
**TCM**	**289**	**114**	**22.8 ± 1.59**	**39.44** ± 1.40^**b**^	**67**	**13.40 ± 1.12**	**23.18 ± ** **2.15**^**a**^	**60**	**12 ± 0.83**	**20.76 ± ** **1.30**^**a**^	**48**	**9.60 ± 1.69**	**16.60**^**a**^** ± ** **2.06**^**a**^
**TCM + Mel**	**234**	**143**	**28.60 ± 2.37**	**61.11** ± 4.97^**a**^	**44**	**8.80 ± 2.37**	**18.80 ± ** **3.82**^**b**^	**30**	**6 ± ** **1.73**	**12.82 ± ** **2.69**^**b**^	**17**	**3.40 ± 0.24**	**7.26 ± ** **1.07**^**b**^

^a,^^b^Means denoted within the same column with different superscripts are significantly different at (*P* < 0.05)

#### Effect of melatonin addition in in-vitro maturation media on nuclear maturation rate (1^st^ Pb) of matured buffalo oocytes

The maturation rate of buffalo oocytes was significantly (*P* < 0.05) higher in the TCM + Mel group (85.47%) when compared with the TCM group (68.85%). The buffalo oocytes without polar bodies matured in the TCM group showed a significant increase (*P* < 0.05) (14.18%) when compared with oocytes cultured in the TCM + Mel group (6.83%). The degenerated oocytes showed a significant increase in the TCM group (16.95%) than TCM + Mel (7.69%) (Table 2).

**Table 2:** Effect of melatonin addition to maturation media on nuclear maturation rate of buffalo oocytes

**Media**	**No. oocytes**	**1**^**st**^**polar body**			**Without polar body**			**Degenerated**		
		**No.**	**Mean** ** ± SE**	**%**	**No.**	**Mean** ** ± SE**	**%**	**No.**	**Mean** ** ± SE**	**%**
**TCM**	**289**	**199**	**39.8 ± 2.03**	**68.85 ± ** **1.15**^**b**^	**41**	**8.20** ** ± 1.06**	**14.18 ± ** **1.35**^**a**^	**49**	**9.80** ** ± 0.66**	**16.95 ± ** **0.31**^**a**^
**TCM + Mel**	**234**	**200**	**40 ± 4.95**	**85.47 ± ** **1.62**^**a**^	**16**	**3.20** ** ± 0.49**	**6.83 ± ** **0.66**^**b**^	**18**	**3.60** ** ± 0.4**	**7.69 ± ** **1.09**^**b**^

^a,^^b^Means denoted within the same column with different superscripts are significantly different at (*P* < 0.05)

#### Effect of melatonin on developmental competence of in vitro matured buffalo oocytes

The fertilization rate, cleavage rate, and transferable embryo rate of in-vitro matured oocytes were significantly higher (*P* < 0.05) in TCM + Mel 84.21%, 89.58%, 48.83% respectively) when compared with TCM (79.88%,75.55%, and 37.25% respectively), (Table 3).

**Table 3:** Effect of melatonin on developmental competence of in vitro matured buffalo oocytes

**Media**	**Oocytes**	**Fertilization rate**			**Cleavage rate**			**Morula & Blastocyst**		
	**No.**	**No.**	**Mean ± ** **SE**	**%**	**No.**	**Mean ± ** **SE**	**%**	**No. Morphologically abnormal oocytes**	**Mean ± ** **SE**	**%**
**TCM**	169	135	27 ± 7.15	79.88 ± 2.70^b^	102	20.4 ± 5.64	75.55 ± 2.49^**b**^	38	7.60 ± 2.44	37.25 ± 2.01^**b**^
**TCM + Mel**	171	144	28 ± 4.45	84.21 ± 2.15^**a**^	129	25.8 ± 4.18	89.58 ± 0.86^**a**^	63	12.60 ± 2.22	48.83 ± 1.15^**a**^

^a,^^b^Means denoted within the same column with different superscripts significantly differ at (*P* < 0.05)

### Experiment 2: effect of melatonin on morphological changes of vitrified/ warmed in-vitro matured buffalo oocytes

Table 4 showed no significant differences between the percentages of recovered in-vitro matured buffalo oocytes after thawing in vitrified groups.

**Table 4:** Effects of the melatonin on the viability and morphology of the *in*-*vitro* matured vitrified/ warmed buffalo oocytes

Media	No. of mature oocytes No.	Recovered oocytes after warming No. (%)	Morphologically normal oocytes No. (%)	Morphologically abnormal oocytes No. (%)	X^2^ (P)
VTCM	176	166 (94.31)^**a**^	112 (67.46)^**c**^	54 (32.53)^a^	11.81 (0.01)
VTCM + Mel	182	176 (96.70)^**a**^	128 (72.72)^**b**^	48 (27.27)^**b**^	
VS2 + Mel	168	160 (95.23)^**a**^	134 (83.75)^**a**^	26 (16.25)^**c**^	

*X*^*2*^ chi-square value, *pp* value

^a,^^b,c^Means denoted within the same column with different superscripts are significantly different at (*P* ≤ 0.01)

There were significant differences (*P* ≤ 0.01) between the percentages of normal in-vitro matured vitrified buffalo oocytes after warming in VTCM, VTCM + Mel, and VS2 + Mel groups (67.46%, 72.72%, and 83.75%, respectively). There were significant differences between the percentages of morphologically abnormal in in-vitro matured vitrified buffalo oocytes after warming in VTCM, VTCM + Mel, and VS2 + Mel groups (32.53%, 27.27%, 16.25%, respectively) (Table 4).

The morphological abnormality of vitrified oocytes with leakage of cellular content and fragmented cytoplasm was significantly higher in the VTCM + Mel group (31.25% and 25%, respectively) than in the VTCM group (22.22% and 9.26%, respectively) and VS2 + Mel group (23.1% and 11.53%). The percentage of vitrified oocytes with cracking of zona pellucida and zona rapture was significantly higher in the VTCM group (25.93% and 14.81%, respectively) than inVS2 + Mel (19.23% and 7.69%, respectively) and VTCM + Mel group (10.42% and 8.33%., respectively). The Percentage of oocytes with shrinkage of cytoplasm was significantly higher in theVS2 + Mel group (38.45%) than VTCM and VTCM + Mel groups (27.78% and 25%, respectively) (Table 5). The abnormality of matured oocytes was significantly (*P* < 0.01) higher in shrinking in the cytoplasm, cracking of zona pellucida, and leakage of cellular content when compared with fragment cytoplasm and zona rapture in the VTCM group. The leakage, shrinking, and fragment of the cytoplasm were significantly higher than the cracking and rupture of the zona pellucida of oocytes in the VTCM + Mel group. Moreover, the shrinking of cytoplasm, cracking of zona pellucida, and leakage of cellular content are significantly higher than fragmented cytoplasm and zona rapture in the VS2 + Mel group.

**Table 5:** Types of abnormalities of vitrified/warmed in-vitro matured buffalo oocytes

**Media**	**No. of abnormal oocytes**	**Leakage of cellular content** **N** **(%)**	**Cracking of zona pellucida** **N** **(%)**	**Shrinkage of cytoplasm** **N** **(%)**	**Fragmented** **N** **(%)**	**Zona rapture** **N** **(%)**	**X**^**2**^**(p)**
VTCM	54	12 (22.22%)^b^**	14 (25.93%)^a^**	15 (27.78%)^b^**	5 (9.26%)^b*^	8 (14.81%)^a*^	22.41 (0.01)
VTCM + Mel	48	15 (31.25%)^a^**	5 (10.42%)^c^*	12 (25%)^b^**	12 (25%)^a^**	4 (8.33%)^b^*	
VS2 + Mel	26	6 (23.1%)^b^**	5 (19.23%)^b^**	10 (38.45%)^a^***	3 (11.53%)^b*^	2 (7.69%)^b^*	

*X*^*2*^ chi-square value, *p p* value for comparing between vitrified groups

^***, **, *^ Subscript significance difference within the same raw (*P* < 0.01)

^a,^^b,c^ Means denoted within the same column with different superscripts are significantly different at (*P* < 0.01)

### Experiment 3: effect of melatonin on developmental competence of in-vitro matured vitrified/warming buffalo oocytes

**Fig. 1:**
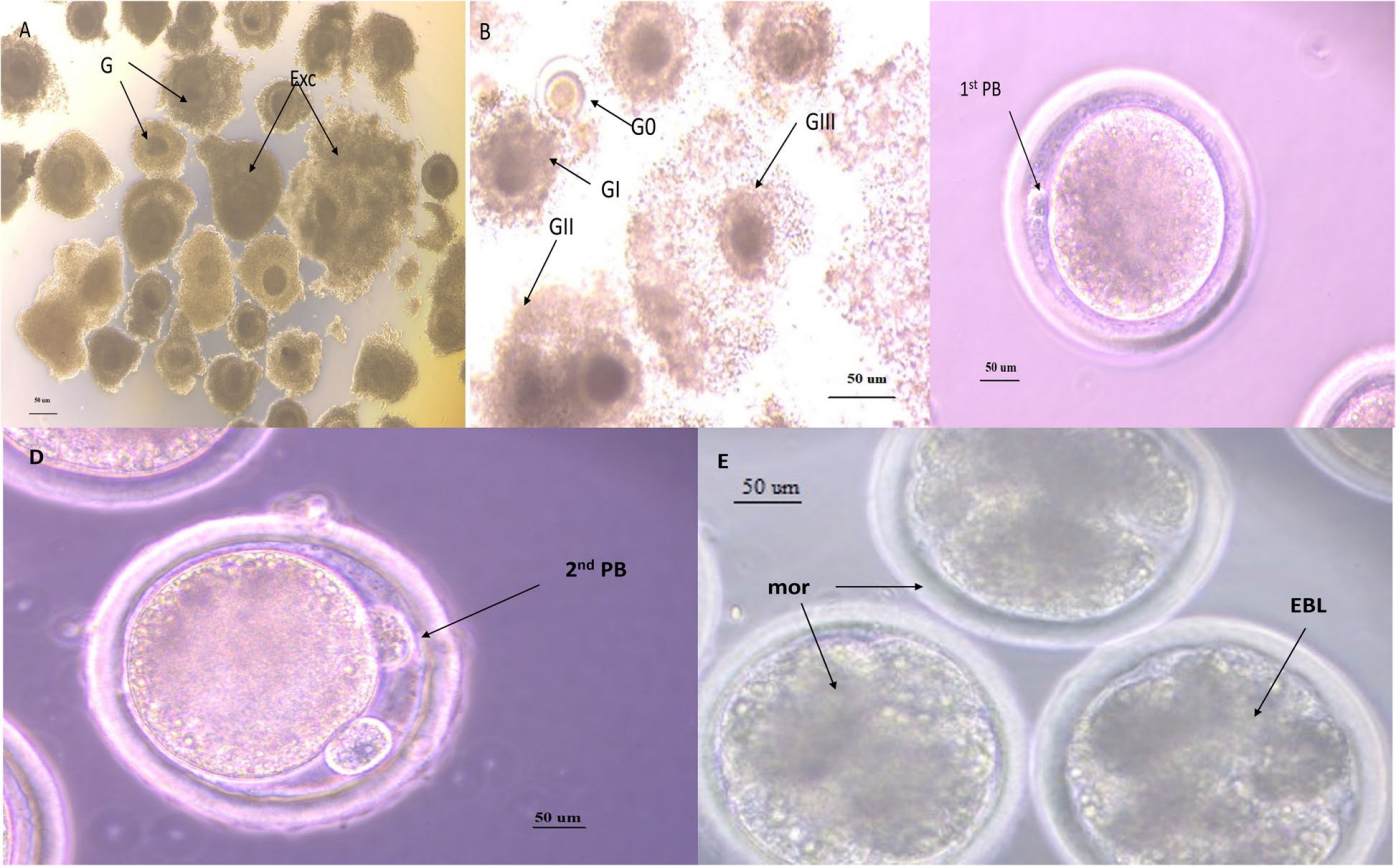
Buffalo oocyte quality and oocyte developmental competence using inverted microscope Zeiss using magnification 20X, **A** showed oocyte quality G = good, Exc = Excellent, **B** cytoplasmic maturation GIII = full expansion, GII = Modred expansion, GI = low expansion, **C** nuclear matured oocytes, 1st PB = 1^st^ polar body, **D** Fertilized oocytes 2^nd^ PB = 2nd polar body, **E** transferable embryo, mor = morula, EBL = early blastocyst

The fertilization rate showed no significant differences between the VTCM group (82.14%), VTCM + Mel group (89.06%), and VS + Mel group (85.07%). The cleavage rate and transferable embryos rate of vitrified /warmed in-vitro matured buffalo oocytes was significantly (*P* < 0.01) increased in VTCM + Mel (63.20%, 28.20% respectively) and VS2 + Mel groups (61.80%, 27% respectively), when compared with VTCM group (51.40%, 17%, respectively). In contrast, there was no significance between VTCM + Mel and VS2 + Mel in both cleavage and transferable embryo rates (Table 6, Fig. 1).

**Table 6:** Effect of melatonin on developmental competence of in vitro matured vitrified/warmed buffalo oocytes

**Media**	**Normal oocytes**	**Fertilization rate**			**Cleavage rate**			**Morula & Blastocyst**		
	**No.**	**No.**	**Mean ± S.E.**	**%**	**No.**	**Mean ± S.E.**	**%**	**No.**	**Mean ± S.E.**	**%**
**VTCM**	112	92	18.4 ±	82.14 ±	47	9.40 ±	51.40 ±	8	1.60 ±	17 ±
			2.82	3.08^**a**^		1.36	1.07^**b**^		0.24	1.37^**b**^
**VTCM + Mel**	128	114	22.8 ±	89.06 ±	74	14.80 ±	63.20 ±	20	4 ±	28.20 ±
			2.81	2.34^**a**^		2.81	3.48^**a**^		0.54	1.85^**a**^
**VS2 + Mel**	134	114	22.8 ±	85.07 ±	70	14 ±	61.8 ±	19	3.8	27 ±
			1.52	0.73^**a**^		0.7	1.59^**a**^		1.57	1.41^**a**^

^a,^^b^Superscripts to be compared statistically within the same column. Values with different letters are significantly different (*P* < 0.01)

### Experiment 4: effect of melatonin on mitochondrial distribution and intensity of fresh in-vitro matured and vitrified/warming in-vitro matured buffalo oocytes

**Fig. 2:**
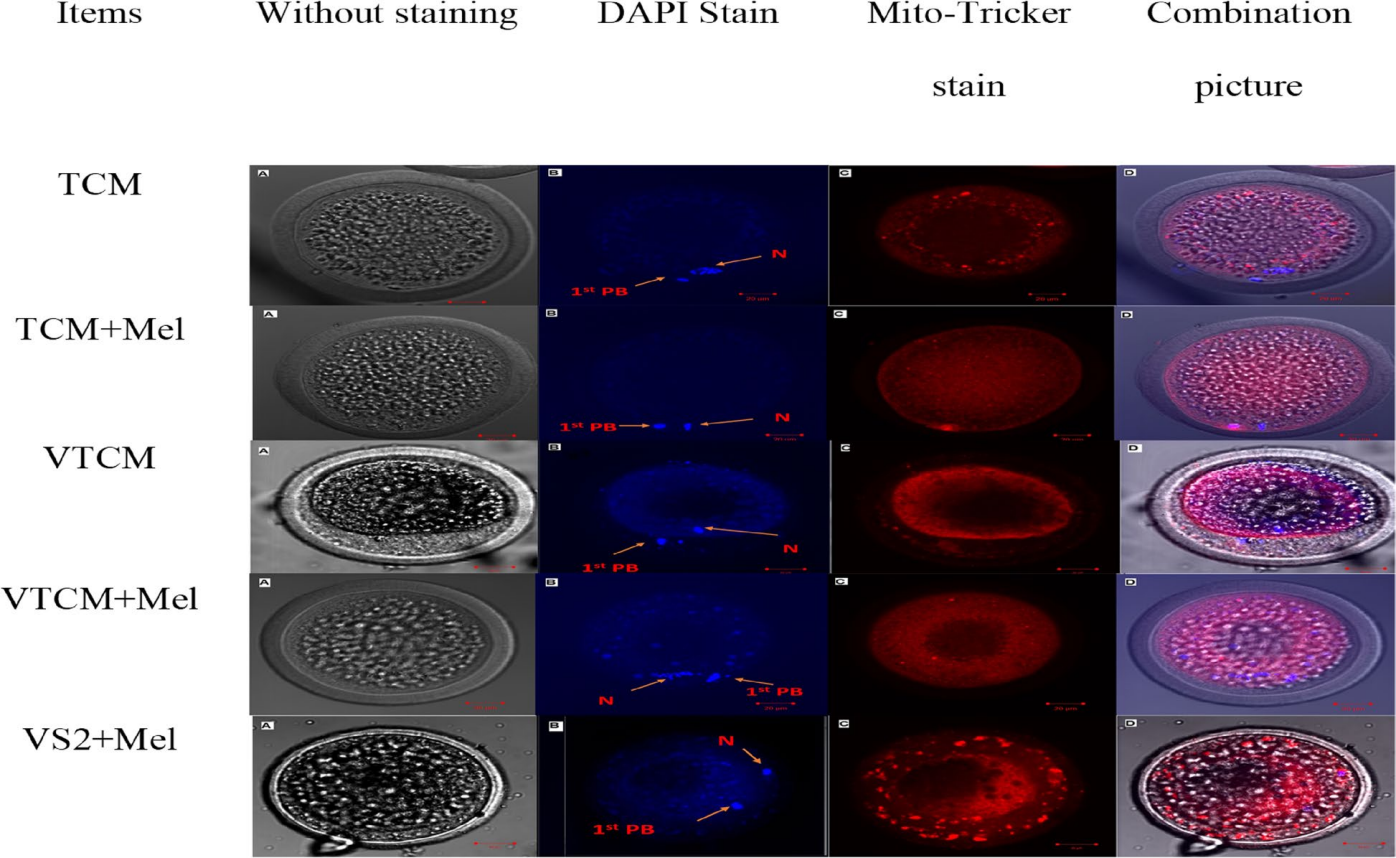
Effect of melatonin addition on in vitro maturation and /or vitrification media on viability and mitochondrial distribution and intensity on in vitro matured fresh and vitrified /thawed buffalo oocytes using confocal microscope zeiss 710 in magnification 200X

More mitochondria diffusely and centrally distributed were significantly increased in the TCM + Mel group (80%, 20% respectively); semi-diffused distribution was significantly higher in the TCM group (20%); peripheral distribution was significantly higher in the VTCM + Mel group (60%); semi- peripheral distribution was significantly higher in VTCM group (70%) (Table 7, Fig. 2). The defused mitochondrial distribution was significantly higher (*P* < 0.01) in the TCM, TCM + mel., VTCM + Mel, and VS2 + Mel than other distributions, while in VTCM the simi-peripheral distribution was significantly higher than other distributions.

**Table 7:** Effect of melatonin on mitochondrial distribution and intensity of in vitro matured and vitrified-warmed in vitro matured buffalo oocytes

**Media**	**No. of oocytes**	**Diffused** **No. (%)**	**semi-diffused** **No. (%)**	**Peripheral** **No. (%)**	**Semi-peripheral** **No. (%)**	**Central** **No. (%)**	**X**^**2**^**(p)**
TCM	30	21 (70%)^**c**, ^^***^	6 (20%)^**a**, ^**	0 (0%)^**d**,^ *	0 (0%)^**c**,^*	3 (10%)^**b**,^^**^	185.65 (0.00)
TCM + Mel	30	24 (80%)^**a**^^,^ ***	0 (0%)^**b**, ^*	0 (0%)^**d**,^^*^	0 (0%)^**c**,^ *	6 (20%)^**a**,^**	
VTCM	30	0 (0%)^**d**,^ *	0 (0%)^**b**,^^*^	9 (30%)^**b**,^ **	21 (70%)^**a**,^ ***	0 (0%)^**d**^	
VTCM + Mel	30	6 (20%)^**d**,^ ***	0 (0%)^**b**^	18 (60%)^**a**^^,^^***^^*^	4 (13.3%)^**b**,^ **	2 (6.7%)^**c**,^^*^	
VS2 + Mel	30	23 (76.7%)^**b**,^ ***	0 (0%)^**b**,^ *	7 (23.3%)^**c**,^**	0 (0%)^**c**, ^*	0 (0%)^**d**,^ *	

*X*^*2*^ chi-square value, *p p* value

^****^, ***, **, *Subscript significance difference within the same raw (*P* < 0.01)

^a,^^b,c,d^Means denoted within the same column with different superscripts are significantly different (*P *≤ 0.01)

The mean number of mitochondrial intensity of fresh mature oocytes in the TCM + Mel group (1698.60) was significantly higher (*P* < 0.01) than the fresh TCM group (456.48) and vitrified/ warmed groups. Among vitrified groups, there was no significant difference in mitochondrial intensity between the TCM + Mel group (327.67) and the VS2 + Mel group (298.47) (Table 8).

**Table 8:** Effect of melatonin on intensity of mitochondria of in vitro matured and vitrified/warmed in vitro matured buffalo oocytes

Media	Mature oocytes	Mean No. of fluorescent intensity ± SE
**TCM**	30	456.48 ± 25.17^**b**^
**TCM + Mel**	30	1698.60 ± 29.92^**a**^
**VTCM**	30	236.75 ± 12.44^**d**^
**VTCM + Mel**	30	327.67 ± 10.98^**c**^
**Vs2 + Mel**	30	298.47 ± 53.42^**c**^

^a,^^b,c,d^Subscript within columns with different letters are significantly different; *p* < 0.01

## Discussion

Melatonin is a hormone that regulates various reproductive functions, including the competence of oocyte development. Melatonin is unique in its hydrophilic and lipophilic properties, allowing it to move quickly throughout all cellular compartments [31]. This means it can be found in the cytosol, nuclei, and mitochondria [32]. Unlike other antioxidants, melatonin has the most desirable characteristics. Its metabolites work together to detoxify radicals [33] and it also stimulates the action of important antioxidant enzymes such as glutathione peroxidase (GSH) and superoxide dismutase (SOD) [34].

The antioxidant melatonin shows promise in boosting the development of oocytes. Our study has revealed that melatonin can improve buffalo oocytes' maturation rate and developmental potential. We observed a significant difference in cumulus expansion and nuclear maturation between the oocytes cultured in the TCM group and those treated with TCM + Mel. Such findings are consistent with previous studies that have demonstrated the ability of melatonin to protect oocytes from oxidative stress and enhance their maturation process. Moreover, melatonin has been reported to promote the maturation of porcine oocytes [35], buffalo [13, 36], mice [28], bovine [37], ovine [38], and humans [39]. Melatonin has been found to improve cytoplasmic maturation in bovine oocytes by returning organelles to their proper locations, increasing the levels of intracellular GSH and ATP, activating genes responsible for oocyte maturation [37, 40], and enhancing antioxidant capacity in mouse eggs [41] Additionally, melatonin can regulate gene expression in both oocytes and cumulus cells [35], studies have shown the positive effects of melatonin on the development of blastocysts in various animals, including bovine [37], sheep [42] (porcine [35], mice [28], buffalo [13, 36], goat [43]), and even human [44]. However, some studies have suggested that melatonin may not be as effective in bovine, as adding it to an IVM medium did not result in higher cleavage or blastocyst rates [45]. These differing results may be due to factors such as the dosage, supplier, or batch of melatonin used for IVM. Melatonin has been found to improve the developmental ability of low-quality oocytes. The goats treated with melatonin showed a higher growth rate to the blastocyst stage than untreated oocytes [46]. Additionally, melatonin enhances preantral follicle pig oocytes' developmental competence [47] and cleavage and blastocyst rate of inferior quality bovine oocytes [48]. Melatonin also helps to lower the levels of ROS in oocytes [49, 50], safeguarding them from the negative effects of oxidative stress and preventing meiotic spindle changes [51], DNA damage [52], apoptosis [53], endoplasmic reticulum stress [54], and aging [55]. Furthermore, it increases the antioxidant capacity in mouse oocytes [35].

This study is the first to explore the impact of melatonin on the physical changes of vitrified/thawed in vitro matured buffalo oocytes. The findings show that vitrification with 10-9M melatonin resulted in significant differences (*p* ≤ 0.01) between the VTCM + Mel, VS2 + Mel, and VTCM groups. The VTCM + Mel group had a high percentage of vitrified oocytes with leaked cellular content and fragmented cytoplasm (31.25% and 25%, respectively). The VTCM group had a high percentage of vitrified oocytes with cracking of zona pellucida and zona rapture (25.93% and 14.81%, respectively). The VS2 + Mel group had a high percentage of oocytes with cytoplasmic shrinkage (38.45%). Ismail's [16] previous research indicated that vitrification of buffalo oocytes significantly affected their morphology. Melatonin enhanced the normal physical changes of vitrified buffalo oocytes, in agreement with previous reports on mouse oocytes. It is important to note that mammalian oocytes are very sensitive to temperature and osmotic pressure changes due to their complex subcellular structure [56]. Cryopreservation can cause significant damage to both oocytes and embryos, with the oocyte membrane being the primary site of damage [57]. Vitrification can lead to hardening of the ZP of oocytes, which can significantly impact their ability to develop [57]. However, studies have shown that melatonin, a potent antioxidant and free radical scavenger, can help reduce oocyte ROS levels. This has been observed in pigs [58], buffalo [13], bovine [59], and camel [10] species. Additionally, melatonin helps maintain adequate levels of endogenous antioxidant enzymes [60] and boosts the level of the antioxidant glutathione (GSH) [61].

After undergoing the process of vitrification and thawing, the oocytes experienced a decrease in GSH synthesis [62] and an increase in ROS production, leading to a reduction in their developmental competence [62].

However, the addition of melatonin has been found to enhance the development of vitrified/thawed oocytes during in vitro development [56]. Recent studies have shown that melatonin supplementation can improve the developmental competence of oocytes in buffalo, and this positive impact is attributed to its ability to reduce oxidative stress [63]. Further research has demonstrated that ROS detoxification is crucial in restoring embryo metabolic functions after thawing [64]. Adding melatonin to maturation, culture, and vitrification media has been found to significantly influence the developmental competence of bovine oocyte and embryo vitrification procedure [65]. Adding 10^–7^ mol/L melatonin to the vitrification-warming and in vitro maturation media showed a significant increase in the maturation rate for mouse GV oocytes [66]. Adding melatonin to in vitro maturation media also improved the meiotic maturation of mouse vitrified oocytes [27]. Moreover, melatonin improved the in vitro survival rate of mouse preantral follicles after vitrification-thawing [67]. Additionally, it enhanced the developmental potential of parthenogenetically activated embryos produced from vitrified mature oocytes in mice [68]. The developmental competence of vitrified-warmed mouse oocytes can be improved by adding melatonin (10^–9^ mol/L) during warming, recovery, parthenogenetic activation, and in vitro culture of embryos. This improvement is mainly due to the modulation of oxidative stress, improvement of mitochondrial function, regulation of spindle assembly and chromosome arrangement, and inhibition of apoptosis [18]. However, melatonin may not always be effective in improving the development of cryopreserved metaphase II mouse oocytes, as some studies have shown. This may be due to varying concentrations and culture conditions of melatonin [69]. This study found that a higher percentage of oocytes in the TCM + Mel group (80%) had a diffuse distribution of mitochondria throughout the cytoplasm compared to other groups. This result is consistent with Ismail's findings [16]. The peripheral distribution of mitochondria was significantly higher in the vitrified VTCM + Mel group (60%), while the semi-peripheral distribution was significantly higher in the vitrified VTCM group (70%). The fresh TCM + Mel group showed significant central distribution (20%). The distribution of mitochondria in mature oocytes is usually homogeneous throughout the cytoplasm, providing equal distribution of mitochondria between zygote cells after fertilization [70]. It has also been shown that a higher density and distribution of active mitochondria is correlated with faster nuclear maturation [71]. Antioxidants added to the maturation media can enhance oocyte mitochondrial activity. Previous studies have demonstrated that melatonin acts as a protective agent that can promote oocyte development [72]. Melatonin enhances oocyte mitochondrial function, increases ATP production [73], and reduces mitochondrial DNA damage [11]. In addition, studies show that melatonin can have positive effects on goats [74] and bovine oocytes. It lowers intracytoplasmic ROS levels, increases mitochondrial activity, and boosts ATP content [37]. Melatonin increases mitochondrial function after an in vitro culture of sheep's secondary follicles. To be more precise, bovine oocytes treated with melatonin had higher normal distribution rates of mitochondria than untreated oocytes [49]. Melatonin addition also increases the rates of oocytes with dispersed mitochondria in the cytoplasm. Furthermore, it reduces mitochondrial oxidative stress in mouse embryos [75] and can potentially improve the quality of human oocytes [11]. Melatonin has been found to be beneficial in enhancing the developmental competence of cryopreserved oocytes. This is achieved through the reduction of oxidative stress and protection of mitochondrial function. Melatonin also indirectly promotes the meiotic competence of oocytes while decreasing the production of ROS during oocyte maturation [76]. Melatonin is a crucial substance that helps improve the quality of oocyte vitrification and reduces oxidative stress imbalance in PCOS patients, thus maintaining oocyte mitochondrial function [17]. Studies have shown that the process of vitrification and warming can affect the intracellular oxidative status, leading to an increase in ROS activity that may harm the structure, distribution, and function of mitochondria in oocytes [77]. In buffalo, mitochondria are arranged in the cortical region [78], which is the first site to be damaged during freezing [79]. Moreover, damage to oocyte mitochondria during vitrification procedures may occur with or without visible morphological changes [80]. The distribution and intensity of mitochondria can impact the cytoplasmic and nuclear maturation of oocytes [81]. Melatonin is a potent antioxidant that specifically targets mitochondria [65]. In a recent study, the group treated with melatonin showed significantly higher mitochondria intensity than the other groups. These results are consistent with Kandil's [10] findings that mitochondrial intensity was significantly higher in melatonin-treated oocytes of camels.

## In conclusion

Adding 10 ^−9^M melatonin to the maturation or vitrification medium can enhance the in vitro developmental capacity of buffalo oocytes, and increase the viability, mitochondrial distribution, and intensity of fresh and vitrified thawed buffalo oocytes.

### Supplementary Information

**Supplementary Material 1.**

## Data Availability

Not applicable.
