# Supplementary material for: Effect of melatonin on developmental competence, mitochondrial distribution, and intensity of fresh and vitrified/thawed in vitro matured buffalo oocytes
Source: Reprod Biol Endocrinol. 2024 Apr 5;22:39. doi: 10.1186/s12958-024-01209-7 (PMC10996257; doi:10.1186/s12958-024-01209-7)
Supplement: Supplementary file 1 — Supplementary Material 1. [file 12958_2024_1209_MOESM1_ESM.pdf]

Manuscript Title: Effect of melatonin on developmental competence, mitochondrial distribution, and intensity of fresh and vitrified /thawed *in vitro* matured buffalo oocytes

Additional File 1

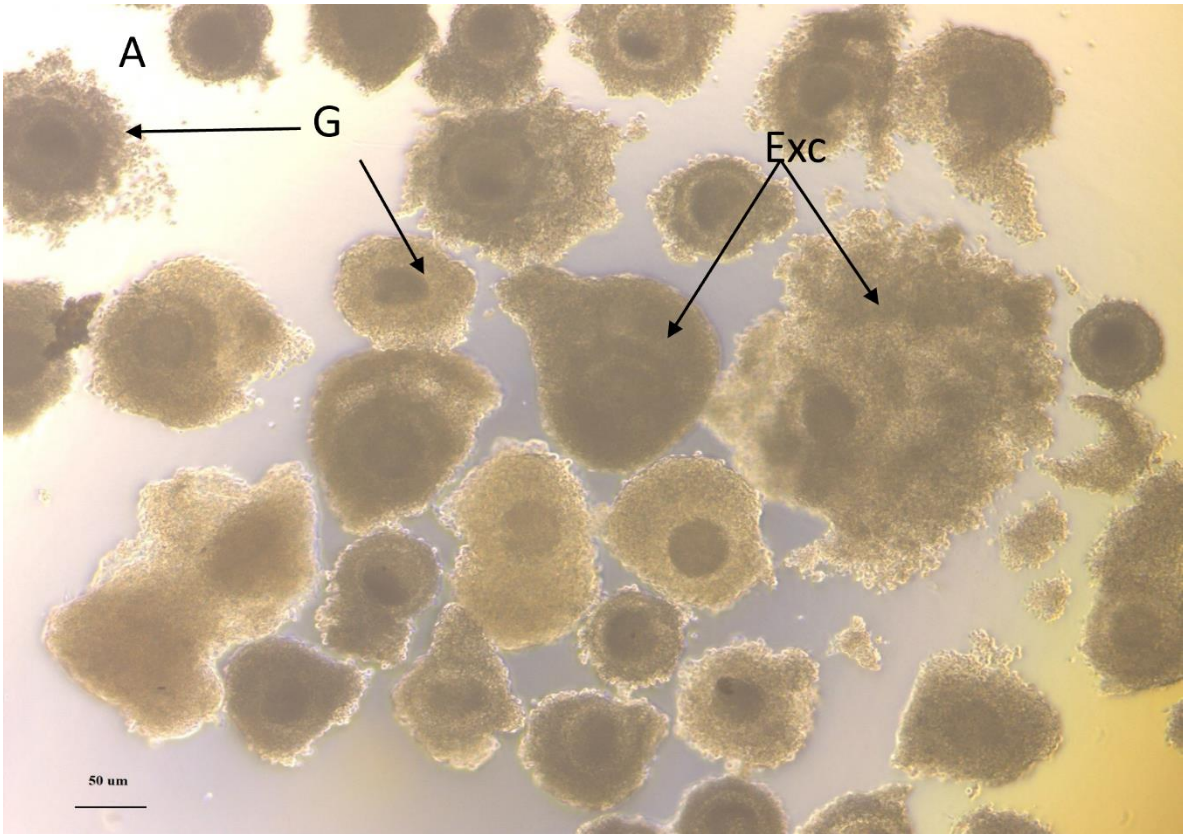

Fig 1 A, Buffalo's immature oocytes quality using inverted microscope Zeiss using magnification 20X. G= Good oocytes, Exc= Excellent oocytes

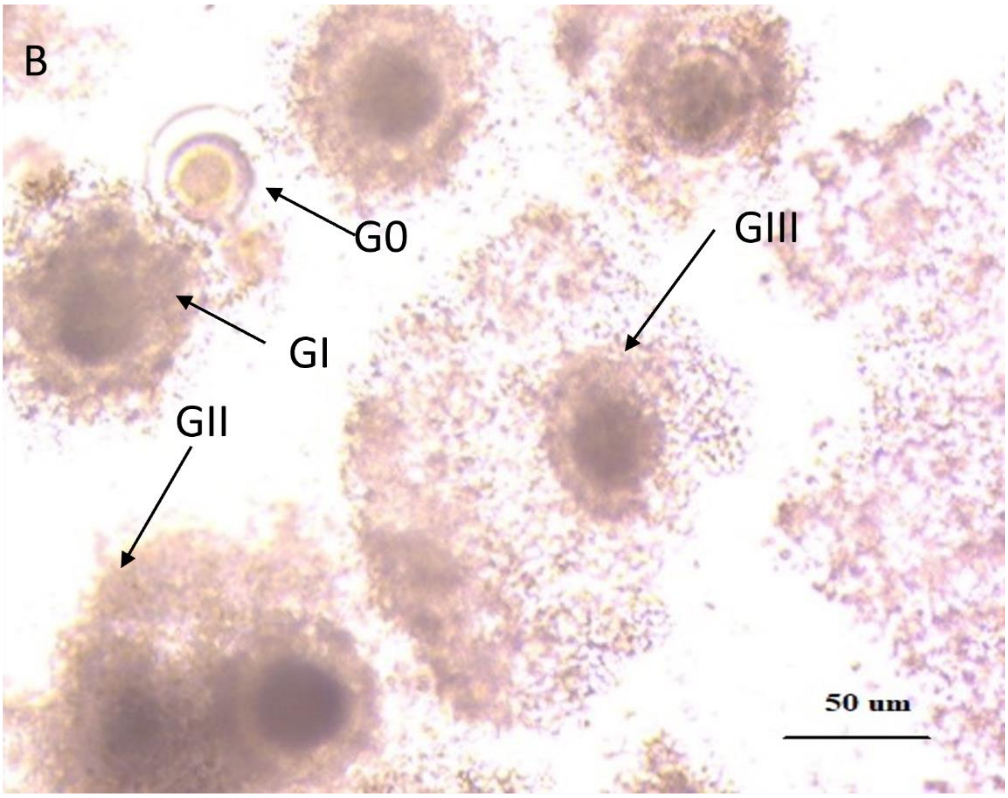

Fig1 B, Cytoplasmic maturation of buffalo's in vitro matured oocytes using inverted microscope Zeiss using magnification 20X, GIII= full cumulus expansion, GII= Modred cumulus expansion, GI= little cumulus expansion, and G0= No cumulus expansion

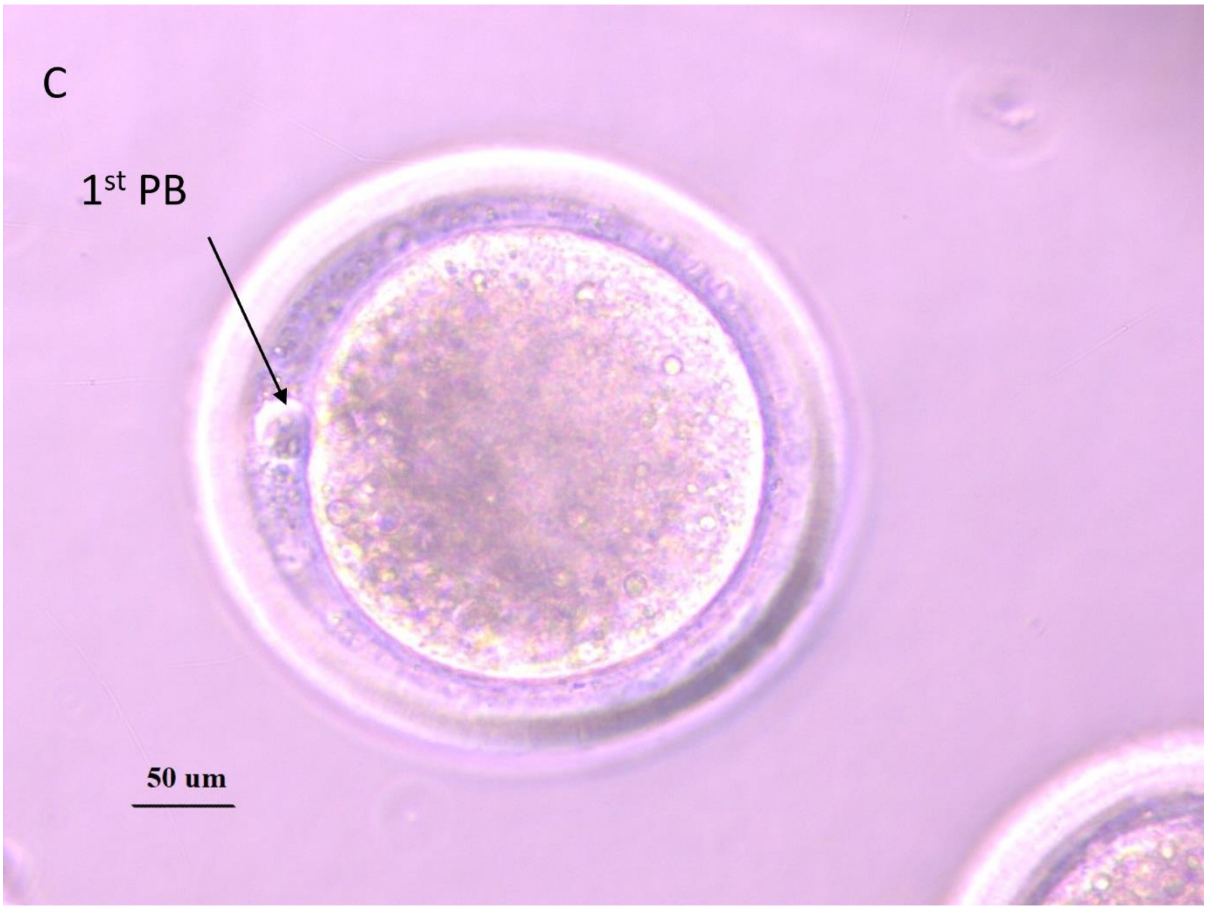

Fig 1 C, Nuclear maturation of in vitro matured buffalo oocytes using inverted microscope Zeiss using magnification 20X, 1st PB= first Polar Body

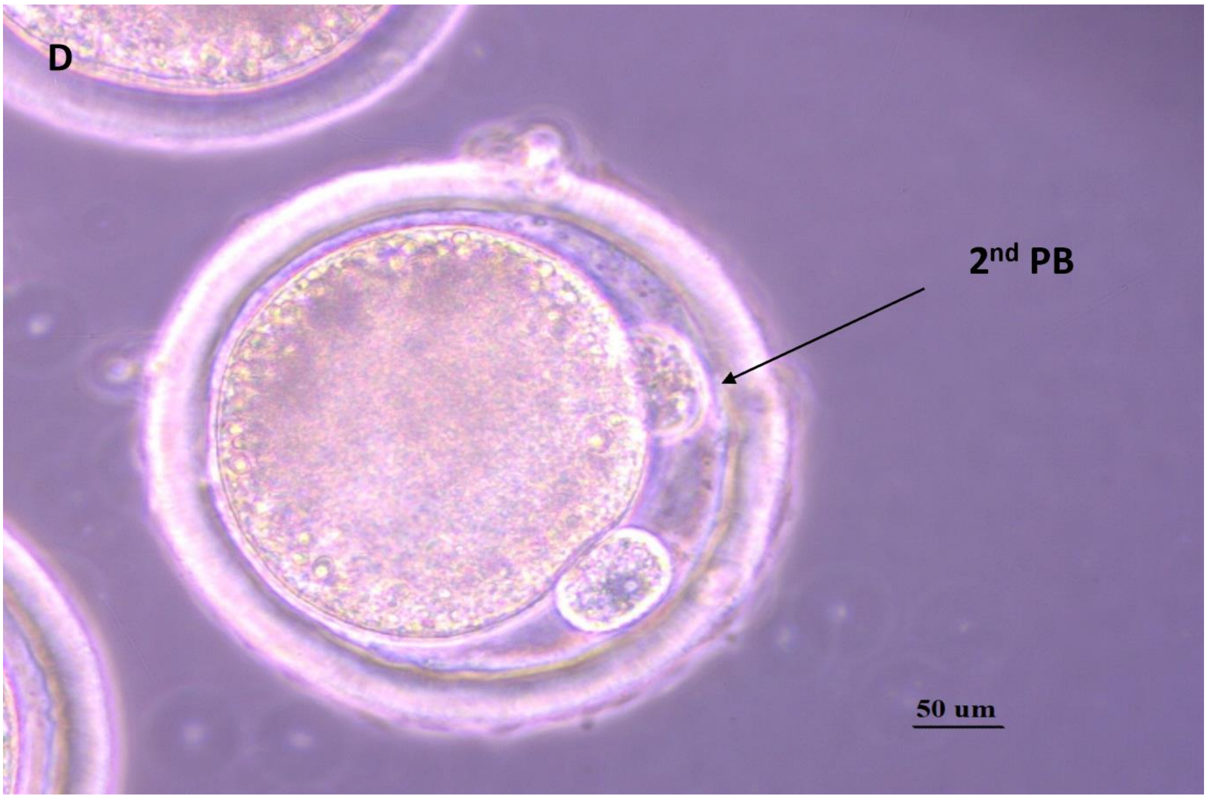

Fig1 D, In-vitro fertilized buffalo's oocytes using inverted microscope Zeiss using magnification 20X, 2nd PB= second Polar Body

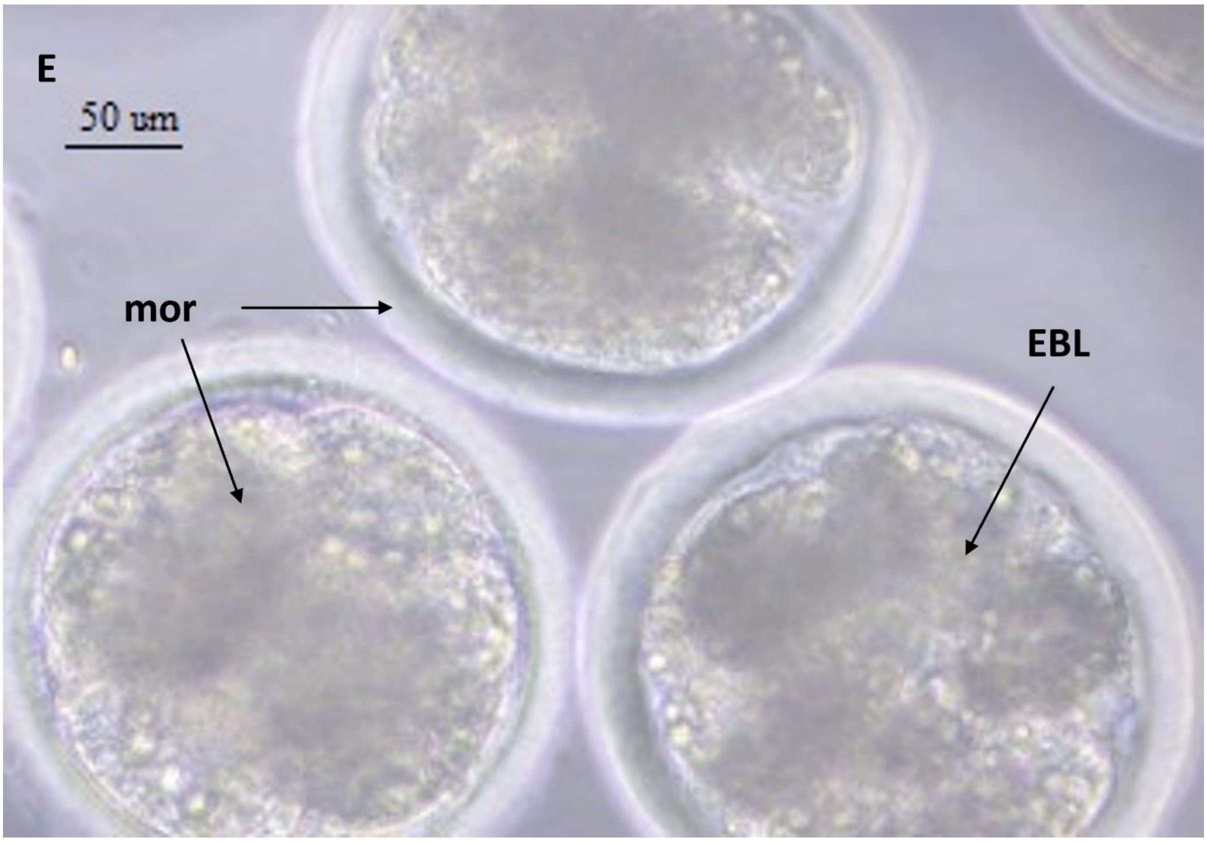

Fig1 D, In-vitro produced buffalo's transferable embryos using inverted microscope Zeiss using magnification 20X, mor= morula and EBL= Early Blastocyst

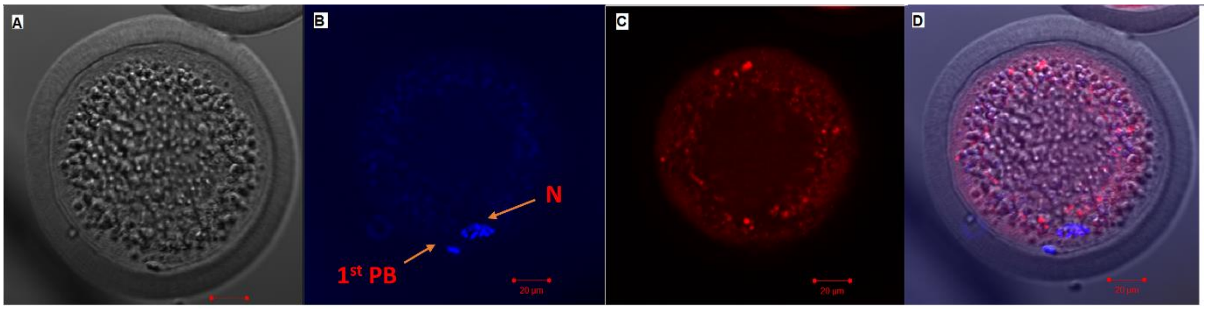

Fig 2 TCM, In vitro matured buffalo oocyte in TCM control fresh oocytes A) Without Stain B) Stained with DAPI showed 1st polar body (1st PB) and reminder nucleus (N) C) Mitochondria stained with Mito Tricker Red stain showed semidifused mitochonderial distribution D) The combination picture of (A+B+C) using confocal microscope Zeiss 710 in magnification 200X.

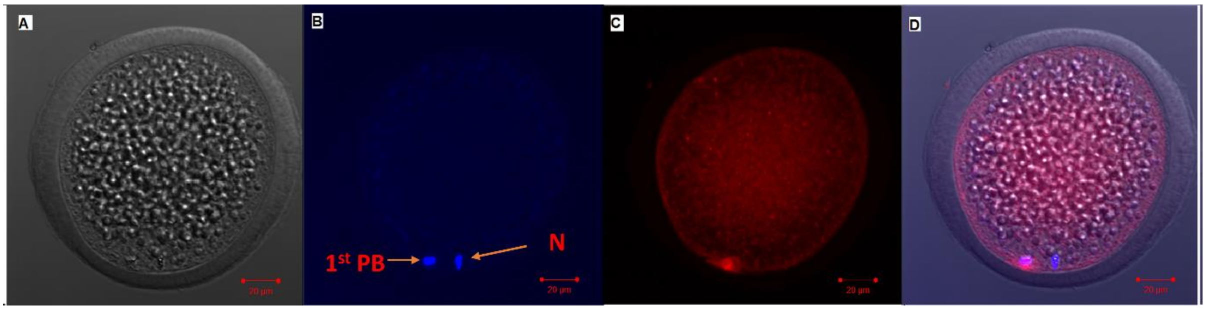

Fig 2 TCM+Mel, In vitro matured buffalo oocyte in TCM-199 medium + Melatonin fresh oocytes A) Without Stain B) Stained with DAPI showed 1st polar body (1st PB) and reminder nucleus (N) C) Mitochondria stained with Mito Tricker Red stain showed difused mitochonderial destreibution D) The combination picture of (A+B+C) using confocal microscope Zeiss 710 in magnification 200X.

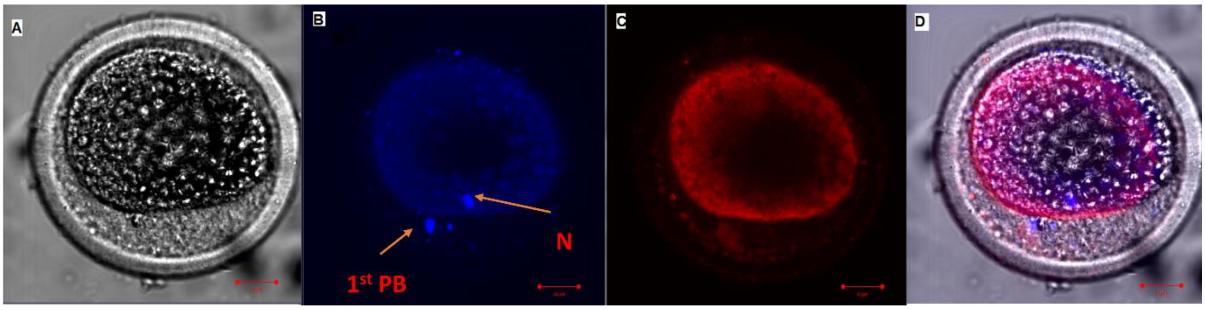

Fig 2 VTCM, In vitro matured buffalo oocyte in TCM media vitrified/ warmed oocytes A) Without Stain B) Stained with DAPI showed 1st polar body and reminder nucleus C) Mitochondria stained with Mito Tricker Red stain showed preferal mitochonderial distribution D) The combination picture of (A+B+C) using confocal microscope Zeiss 710 in magnification 200X.

.

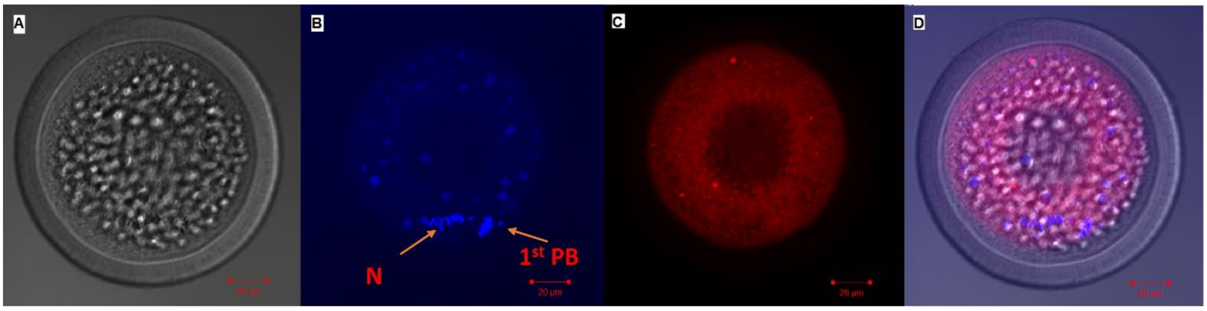

Fig 2 VTCM+Mel, In vitro matured buffalo oocyte in TCM media + Melatonin vitrified/ warmed oocytes A) Without Stain B) Stained with DAPI showed 1st polar body and reminder nucleus C) Mitochondria stained with Mito Tricker Red stain showed semiprepheral Mitochonderial distribution D) The combination picture of (A+B+C) using confocal microscope Zeiss 710 in magnification 200X.

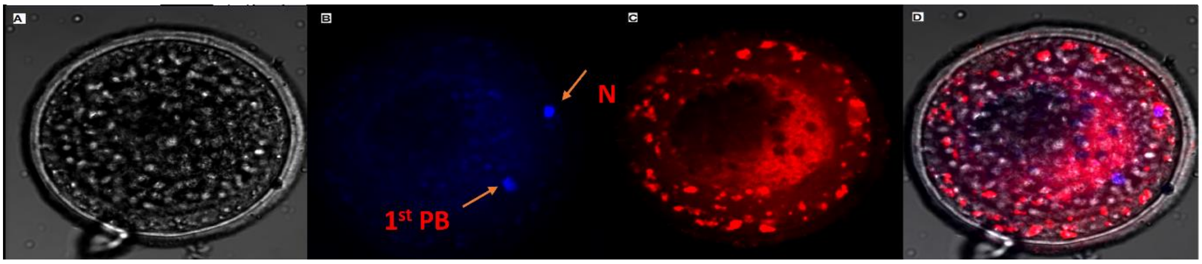

Fig 2 VS2+Mel, In vitro matured buffalo oocyte in TCM media and vitrified in VS2 +Melatonin/ warmed oocytes A) Without Stain B) Stained with DAPI showed 1st polar body and reminder nucleus C) Mitochondria stained with Mito Tricker Red stain showed difused mitochonderial distribution D) The combination picture of (A+B+C) using confocal microscope Zeiss 710 in magnification 200X.
